# Newcastle disease virus induces degradation of folliculin to regulate host cell energy metabolism and facilitate viral replication^☆^

**DOI:** 10.1016/j.psj.2025.105314

**Published:** 2025-05-23

**Authors:** Mengqing Yang, Xianjin Kan, Xinna Li, Yingjie Sun, Ying Liao, Xusheng Qiu, Lei Tan, Cuiping Song, Chan Ding

**Affiliations:** a Department of Avian Infectious Diseases, Shanghai Veterinary Research Institute, Chinese Academy of Agricultural Science, Shanghai, China; b School of Agriculture and Biology, Shanghai Jiao Tong University, Shanghai, 200240, China; c Jiangsu Co-innovation Center for Prevention and Control of Important Animal Infectious Diseases and Zoonosis, Yangzhou University, Yangzhou 225009, Jiangsu Province, China

**Keywords:** NDV, FLCN, SREBP1c, De novo fatty acid synthesis, Energy metabolism

## Abstract

Viral replication is an energy-intensive process that often induces energy stress in host cells, and efficient mobilization of host cell energy resources facilitates optimal viral replication. The mechanisms by which Newcastle disease virus (NDV) regulates the host energy metabolism to facilitate its replication remain incompletely understood. For this purpose, transcriptomic analysis was conducted to delineate the transcriptional changes during NDV infection. The results demonstrated that NDV infection downregulated the transcriptional levels of enzymes associated with de novo fatty acid synthesis. Subsequent investigations demonstrated that the active form of the sterol regulatory element-binding protein 1c (SREBP1c) a master transcription factor governing lipid biosynthesis pathways, exhibits reduced expression following viral infection. Notably, SREBP1c activation is negatively regulated by folliculin (FLCN), a tumor suppressor protein that undergoes during NDV infection. The *de novo* fatty acid synthesis pathway is an energy-intensive process, and the degradation of FLCN may suppress this pathway to maintain cellular energy homeostasis, thereby supporting viral replication. In summary, our findings demonstrate that NDV facilitates its replication by inducing degradation of FLCN, thereby modulating the host cell energy metabolism.

## Introduction

Viruses are obligatory intracellular microbes that hijack the host metabolic machinery to fulfill their biosynthetic requirements (Goodwin et al., 2015). The fundamental structure of viruses consists of nucleic acid and a protein capsid, with some enveloped viruses also featuring a lipid-based envelope derived from host cells (DeLong et al., 2022). Consequently, substantial energy is required to replenish these components after infection (Nagy and Lin, 2020). Extensive viral replication exhausts host cell energy and disrupts metabolism homeostasis, presenting substantial challenges for both viral replication and cell survival. During infection, most viruses have evolved diverse mechanisms to reprogram the host metabolic system (Sanchez and Lagunoff, 2015) , with such metabolic shifts favoring viral replication over host survival (Thyrsted and Holm, 2021). Therefore, investigating how viruses gain metabolic advantages during infection will provide theoretical support for targeted antiviral therapies.

Fatty acids perform multiple critical functions in cells, including energy storage, membrane biogenesis, and the production of signaling molecules (Currie et al., 2013), all of which are essential during viral infection. Cells replenish fatty acids through diverse pathways, such as *de novo* synthesis mediated by enzymatic cascades (Worthmann et al., 2024), extracellular uptake facilitated by fatty acid-binding proteins and receptors (Mashek and Coleman, 2006), and mobilization via lipid droplet degradation, the primary fatty acid reservoirs (Olzmann and Carvalho, 2019). The involvement of fatty acids in viral replication has been well described. For example, coronaviruses enhance replication by triggering lipolysis of lipid droplets, directing liberated fatty acids into mitochondria for energy production (Yue et al., 2023). Poliovirus reprograms intracellular membranes by degrading lipid droplets to supply fatty acids for phosphatidylcholine synthesis, thereby supporting viral replication (Viktorova et al., 2018). However, the role of *de novo* fatty acid synthesis in viral replication remains poorly understood.

*De novo* fatty acid synthesis is a complex and energy-intensive chemical process, facilitated in mammals by a single large multifunctional enzyme complex. Multiple enzymes play essential roles in fatty acid synthesis. ATP citrate lyase (**ACLY**) bridges glucose and fatty acid metabolism by converting six-carbon citrate into oxaloacetate and two-carbon acetyl-CoA. Acetyl-CoA is then carboxylated by acetyl-CoA carboxylase **(ACC)** to produce malonyl-CoA, which catalyzes the rate-limiting step and functions as the most tightly regulated enzyme in the fatty acid biosynthesis pathway (Chen et al., 2019). The enzymatic activity of ACC can be inhibited by phosphorylation at serine 80 and serine 221 (Lally et al., 2019). Fatty acid synthase (**FASN**) catalyzes sequential condensation of malonyl-CoA and acetyl-CoA to synthesize fatty acids, primarily generating 16-carbon palmitate. Stearoyl-CoA desaturase (**SCD**) introduces double bonds into short-chain fatty acids at the C9 position, converting stearoyl-CoA to oleoyl-CoA. Sterol Regulatory Element-Binding Protein 1c **(SREBP1c)** serves as a key regulator of fatty acid metabolism. (Gosis et al., 2022). Like all SREBPs, SREBP1c exists as a large precursor complex embedded in the endoplasmic reticulum membrane alongside SREBP cleavage-activating protein and insulin-induced gene proteins. Following proteolytic cleavage, the mature N-terminal SREBP1c (nSREBP1c) translocates to the nucleus, where it binds to sterol regulatory elements to activate the transcription of target genes (Wang et al., 2015).

Folliculin **(FLCN)** is a highly conserved protein first identified in 2002 as the causative gene of Birt-Hogg-Dubé syndrome (Menko et al., 2009). It functions as a regulatory protein for transcription factors E3 and EB **(TFE3/TFEB)**. FLCN deficiency selectively inhibits mechanistic target of rapamycin complex 1 (mTORC1)-mediated phosphorylation of TFE3/B, resulting in their nuclear translocation. This nuclear translocation suppresses de novo fatty acid synthesis by preventing the activation of SREBP1c (Gosis et al., 2022). Mitochondrial β-oxidation of fatty acids has been reported to provide energy to support viral replication throughout the viral life cycle (Heaton and Randall, 2010; Yue et al., 2023). Conversely, *de novo* fatty acid synthesis—the anabolic counterpart to β-oxidation—is energetically demanding: the production of a single palmitic acid molecule consumes one acetyl-CoA, seven malonyl-CoA, and 14 NADPH molecules (Paiva et al., 2021). Inhibition of this pathway may conserve cellular energy, potentially facilitating viral replication.

NDV is an enveloped negative-sense RNA virus belonging to the Paramyxoviridae family, recognized as an oncolytic virus that selectively infects various tumor cells (Sitkovskaya et al., 2018, Zhong et al., 2025). As a highly contagious avian-origin pathogen, NDV outbreaks frequently cause substantial economic losses in the poultry industry and is classified as a notifiable disease by the World Organization for Animal Health (Ganar et al., 2014). The mechanism by which NDV manipulates host energy metabolism to support replication remains unclear. In this study, we identified FLCN, a significantly differentially expressed factor following NDV infection, and demonstrated that it facilitates viral replication by modulating host de novo fatty acid synthesis processes.

## Methods

### Cells, viruses and plasmids

The A549 cell line was maintained in F12K medium (Thermo Fisher, 21127030); the HEK-293T, DF-1 and BHK21 cell lines were maintained in DMEM (Thermo Fisher, C11995500BT). Both media were supplemented with 10 % FBS (VivaCell, C2820-0500HI), penicillin at 100 U/mL, and streptomycin at 100 μg/mL (Beyotime, C0222). The NDV strains Herts/33 was procured from the China Institute of Veterinary Drug Control (Beijing, China). The plasmid pHAGE-puro (118692), lentiCRISPR v2 (#52964), psPAX(#12260) and pMD2.G(#12259) were obtained from Addgene. pCMV-HA-FLCN was constructed by inserting the open reading frame (ORF) of FLCN into the vector pCMV-HA.

### Generation of cell lines

To generate *FLCN* knockout (*FLCN*^-/-^) A549 cell lines, HEK-293T cells grown to 80 % confluence in 10 cm dishes were transfected with lentiCRISPR v2 plasmid containing sgRNA (TGCCACTTCTGCGAGCTCCA) (6 μg), psPAX2 (8 μg), and pMD2.G (2 μg) using FuGene (Promega). Lentivirus-containing supernatant was collected 72 hours post-transfection, filtered (0.45 μm), and stored at −80°C. A549 cells grown to 90 % confluence were infected with a 1:1 mixture of F12K medium and lentivirus, followed by replacement with F12K medium with 10 % FBS 24 hours later. Selection with puromycin began 48 hours post-infection and continued for 48 hours, after which surviving cells were expanded and subcloned. For the identification of *FLCN*^-/-^ cell line, Genomic DNA was extracted, and sgRNA-targeted primers (F: TTTGCCAGGTTTGGCTGCAG; R: CAAAAGCTACAGTCAGGATGAGCG) were used for PCR amplification. The product was ligated into the pMD™ 19-T vector (TAKARA) for sequencing.

To generate stable *FLCN* overexpression (*FLCN*^OE^) A549 cell line, pHAGE-FLCN was constructed by inserting the open reading frame (ORF) of FLCN into the lentiviral vector Phage-puro. Lentivirus production and infection were performed following the same protocol used for generating the *FLCN*^-/-^ cell line.

### Pharmacological Inhibitor Studies

A549 cells were infected at an MOI of 1. After removing the inoculum, fresh media containing 2 % FBS and either MG132 (Selleck, S2619), CQ (Sigma, C6628), or vehicle control were added. Cells were incubated for 24 hours.

### Western blot and antibodies

A549 cells were treated as described previously, and lysates were prepared with RIPA buffer, followed by incubation at 100°C for 10 minutes. For nuclear and cytoplasmic fractionation, MOCK or NDV-infected A549 cells were processed using a Beyotime nuclear and cytoplasmic extraction kit (P0027). Protein samples were resolved by 10 % SDS-PAGE, transferred to nitrocellulose membranes, and blocked with 5 % skim milk for 1 hour. Membranes were incubated with primary antibodies overnight at 4°C, followed by secondary antibodies for 2 hours at room temperature. Signals were developed with Western ECL substrate and detected using a Tanon 5200 imaging system; band intensities were quantified via ImageJ.

Primary antibodies included anti-NP (produced in our laboratory), anti-HN (a gift from Zengqi Yang), anti-β Actin, anti-SREBP1c, anti-α-Tubulin, anti-TFE3, anti-Lamin B1 (Proteintech), anti-FLCN, anti-ACC, and anti-p-ACC (Cell Signaling Technology). Secondary antibodies were HRP-conjugated goat anti-mouse and goat anti-rabbit (Abclonal), and Alexa Fluor® 488/594-conjugated goat antibodies (Thermo Fisher Scientific).

### Immunofluorescence

Cells cultured on coverslips were either mock-treated or infected with NDV at an MOI of 1, as described in the figure legends. After incubation, cells were rinsed three times with PBS, fixed with 4 % paraformaldehyde for 10 minutes at room temperature, and permeabilized with 0.5 % Triton X-100 for 10 minutes. Following additional PBS rinses, cells were blocked with 3 % bovine serum albumin (BSA) at 37°C for 30 minutes, then incubated with primary antibodies for 2 hours at 37°C or Lyso Tracker (Beyotime, C1046) for 10 minutes. The cells were then washed three times with PBS and incubated with Alexa Fluor 488- or 594-conjugated secondary antibodies (Thermo Fisher Scientific) at 37°C for 2 hours. Coverslips were mounted with DAPI-containing mounting medium (Beyotime, P0131) or DAPI-free mounting medium (MERCK, F4680), air-dried in the dark at room temperature, and fluorescence images were acquired using Cytation 5 (Biotek).

### Plaque assays

Serial tenfold dilutions of supernatants from infected cells were added 200 μL to BHK-21 cell monolayer and adsorbed for 60 min at 37 °C. Cells were washed, and Methyl cellulose medium was overlaid on the cells and placed at 37 °C. After 48-72 h of incubations, the cell monolayers were stained with crystal violet and plaques were counted.

### RNA isolation and RT-qPCR assay

Total RNA was extracted from mock-treated or NDV-infected cells (MOI=1, 18 h) using TRIzol reagent, and RNA samples (2 μg each) were then reverse transcribed into cDNA using EasyScript One-Step gDNA Removal and cDNA Synthesis SuperMix kit (TransGen Biotech, AE311-03) and HiScript II Q RT SuperMix for qPCR (+gDNA wiper) (Vazyme, R223-01). The resulting cDNA was subsequently used as a template for a Power Green qPCR Mix assay (TransGen Biotech, AQ311). Each sample’s relative mRNA expression levels were normalized to the expression of β-actin mRNA. All samples were assayed three times and were performed in an ABI 7500 real-time PCR system (Applied Biosystems, Waltham, MA, USA). The qRT-PCR primers were designed as follows. Human-specific primers:

*SCD1* (F: GGTGAACAGTGCCGCGCATCTC; R: GTGTGGTGGTAGTTGTGGAAGCC;)

*FASN* (F: GGAGGTGGTGATAGCCGGTAT; R: TGGGTAATCCATAGAGCCCAG;)

*ACACA* (F: ATGGGCGGAATGGTCTCTTTC; R: TGGGGACCTTGTCTTCATCAT;)

*ACLY* (F: CAGCCAAGGCAATTTCAGAGC; R: CTCGACGTTTGATTAACTGGTCT;)

*FLCN* (F: TCTTCAGCATTGTCCGCCAG; R: AGTTGATGAGGTAGATCCGGTC;)

*β-actin* (F: CATCGTGATGGACTCTGGTG; AGGGCAACATAGCACAGCTT;)

*Avian-specific primers:*

*SCD* (F: CGGATGCAGACCCTCACAAT; R: GGGCTTGTAGTATCTCCGCTG;)

*FASN* (F: GAATCCAGAAGGGCCAACGA; R: TCCAAGGGAGCAGCTTTTGT;)

*ACACA* (F: AATGGCAGCTTTGGAGGTGT; R: TTCTGTTTGGGTGGGAGGTG;)

*ACLY* (F: TCACGTACCTCGAGATCAATCC; R: GCAATGTAGGCTTCCTCTGGG;)

*FLCN* (F: AGATATGTGCGAGGGTTGCC; R: CTGAACAGCTGGGGATGGTT;)

*β-actin* (F: TATTGCTGCGCTCGTTGTTGAC; GATACCTCTTTTGCTCTGGGCTTC;)

### Total ATP assay

The ATP assay kit (Beyotime, S0027) was utilized to determine the total ATP levels in cells. A549 cells were seeded in a 6-well plate, infected with NDV at an MOI of 1. At 6, 12, 18 and 24 h p i, cell lysates were collected for analysis. Standard curves were prepared following the manufacturer's protocol, and the ATP content in the cell lysates was quantified accordingly.

### Statistical analysis

Statistical differences were ascertained using GraphPad Prism 8.0 software. Data were expressed as means ± standard deviations. Significance was determined with the two-tailed independent Student’s t-test (*p* < 0.05) between two groups. One-way ANOVA followed by Tukey’s test was used to compare multiple groups (>2).

## Result

### NDV inhibits de novo fatty acid synthesis

**Fig. 1:**
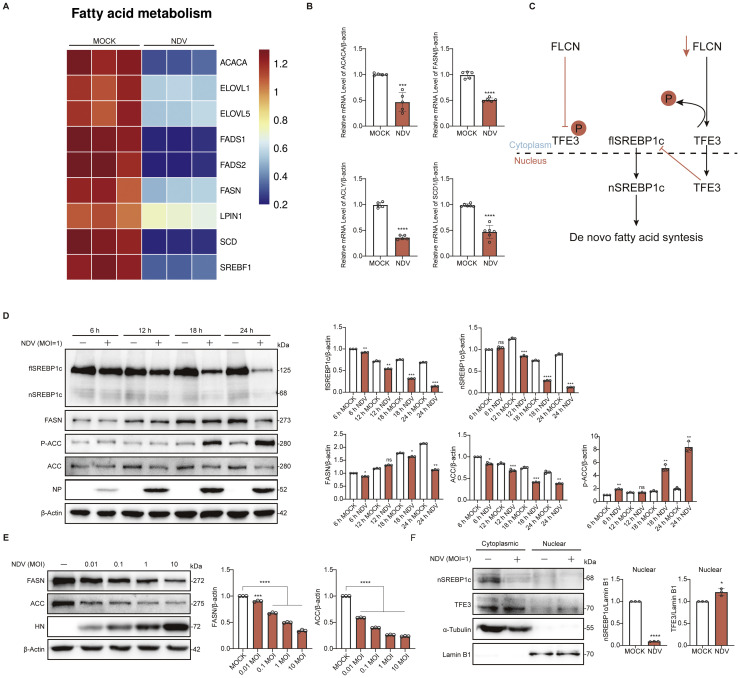
NDV inhibits the *de novo* fatty acid synthesis pathway. A. Differentially expressed enzymes related to *de novo* fatty acid synthesis in the transcriptomic data. B. A549 cells were MOCK- or NDV-infected at an MOI of 1 and RNA was extracted 18 h p i for quantitative PCR analysis. C. FLCN acts as a positive regulator of SREBP1c; its reduction promotes the dephosphorylation of TFE3, enabling TFE3 to function as a transcriptional co-factor that suppresses SREBP1c activation. D. Protein levels of SREBP1c, FASN, ACC and p-ACC at different times points after NDV infection (1 MOI) were assessed by Western blot, with NP serving as a marker for viral infection. E. Protein levels of FASN and ACC at different NDV MOI were assessed by Western blot 18 h p i, with HN serving as a marker for viral infection. F. Nuclear and cytoplasmic fractionation was performed on MOCK or NDV-infected (1 MOI) A549 cells at 18 hours post-infection.

The fatty acid synthesis pathway plays a critical role in the rapid proliferation of cancer cells (Menendez and Lupu, 2007); however, how NDV exploits this pathway remains unclear. Our transcriptomic data revealed a significant downregulation of enzymes involved in de novo fatty acid synthesis following NDV infection (Fig. 1A). The transcriptional levels of selected de novo fatty acid synthesis genes were further validated using quantitative qPCR (Fig 1B and S1A-D). SREBP1c is a key transcriptional regulator of fatty acid synthesis and is positively regulated by FLCN (Fig. 1C). Our results demonstrated that NDV infection led to a marked decrease in both the precursor (flSREBP1c) and active forms (nSREBP1c) of SREBP1c, accompanied by a corresponding reduction in the protein levels of downstream fatty acid synthesis-related enzymes, FASN and ACC (Fig. 1D). Interestingly, following viral infection, not only were the protein levels of de novo fatty acid synthesis enzymes suppressed, but the phosphorylation of ACC, the rate-limiting enzyme of this pathway, was significantly elevated, further inhibiting fatty acid synthesis (Fig. 1D). Additionally, NDV infection alters the protein levels of FASN and ACC in a dose-dependent manner (Fig. 1E). Nuclear-cytoplasmic fractionation experiments also revealed reduced levels of mature SREBP1c and increased nuclear accumulation of TFE3 in virus-infected cells (Fig. 1F). Collectively, these results demonstrated that the *de novo* fatty acid synthesis pathway is inhibited following NDV infection.

### NDV degrades FLCN through the ubiquitin-proteasome pathway

**Fig. 2:**
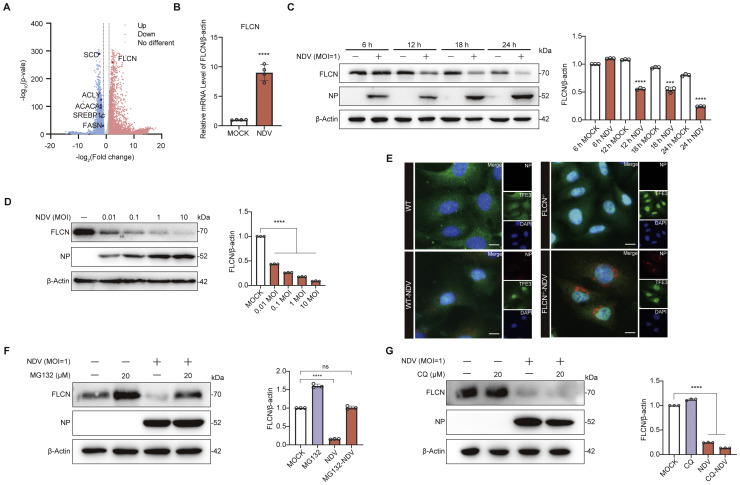
NDV degrades FLCN through the ubiquitin-proteasome pathway. A Volcano plot of FLCN and *de novo* fatty acid synthesis-related factors. Transcriptome study design: A549 cells were infected with NDV (1 MOI) for 18 hours. Each experimental condition (Mock 18 h p i, NDV 18 h p i) consisted of three replicates. B A549 cells were MOCK- or NDV-infected at an MOI of 1 and RNA was extracted 18 h p i for quantitative PCR analysis. C Protein levels of FLCN at different time points after NDV infection (1 MOI) were assessed by Western blot, with NP serving as a marker for viral infection. D Protein levels of FLCN at different NDV MOI were assessed by Western blot 18 h p i, with NP serving as a marker for viral infection. E. Immunofluorescence analysis for colocalization of TFE3 with nucleus. A549 WT and *FLCN*^-/-^ cells were infected with NDV (1 MOI) 18 h p i. Scale bar 10 μm. F and G The effect of MG132 or CQ on FLCN protein levels after NDV infection (1 MOI) was investigated by treating cells with MG132 (10 μM) or CQ (20 μM) simultaneously with viral infection, and cell samples were collected at 18 h p i.

Recent studies have demonstrated that FLCN positively regulates the *de novo* fatty acid synthesis pathway (Fig. 1C) (Gosis et al., 2022). Unexpectedly, the transcriptional level of *FLCN* was significantly upregulated, which contradicts our findings that the *de novo* fatty acid synthesis pathway is inhibited following NDV infection (Fig. 2A-B and S1E). To further investigate this discrepancy, we analyzed the protein levels of FLCN following viral infection. In contrast to its transcriptional upregulation, FLCN protein levels were significantly reduced after NDV infection in a time- and dose-dependent manner (Fig. 2C and D). FLCN inhibits the phosphorylation of TFE3 via mTORC1, thereby preventing its translocation to the nucleus (Li et al., 2022). Consistently, TFE3 was observed to translocate into the nucleus in *FLCN*^-/-^ cells (Fig. 2E). Importantly, we observed nuclear accumulation of TFE3 in WT cells following NDV infection, which is likely a consequence of NDV-induced FLCN degradation (Fig. 2E). These results suggest that FLCN degradation occurs through a post-transcriptional mechanism. To investigate the mechanism of FLCN degradation during NDV infection, MG132 (a proteasome inhibitor) and CQ (a lysosome inhibitor) were used. MG132 treatment effectively reversed virus-induced FLCN degradation, while CQ treatment had little to no impact on FLCN levels, suggesting that the degradation of FLCN is primarily mediated through the proteasome pathway rather than the lysosome. (Fig. 2F and Fig. 2G). These results indicate that FLCN is degraded following NDV infection.

### FLCN inhibits NDV replication

The increase in FLCN transcriptional levels alongside the decrease in protein levels appears to reflect a competitive interplay between viral infection and the host cell. It remains unclear whether FLCN degradation is part of the host antiviral defense mechanism, or a strategy employed by the virus to enhance its replication. To investigate the role of FLCN on NDV replication, we constructed FLCNOE and FLCN-/- cell lines (Fig. S2). In FLCN-/- cell lines, the replication capacity of NDV was enhanced, as evidenced by increased levels of the viral proteins NP and HN within the cells and a higher number of viral particles in the cell supernatant (Fig. 2A and B). In contrast, in *FLCN*^OE^ cell lines, the replication capacity of NDV was suppressed, evidenced by reduced levels of the viral proteins NP and HN within the cells and fewer viral particles in the cell supernatant (Fig. 2C-D). We further verified the inhibitory effect on NDV replication by overexpressing FLCN protein in DF-1 cells (Fig. S3). These findings demonstrate that FLCN negatively regulates NDV replication.

### NDV infection modulates de novo fatty acid synthesis through FLCN and regulates energy homeostasis

**Fig. 3:**
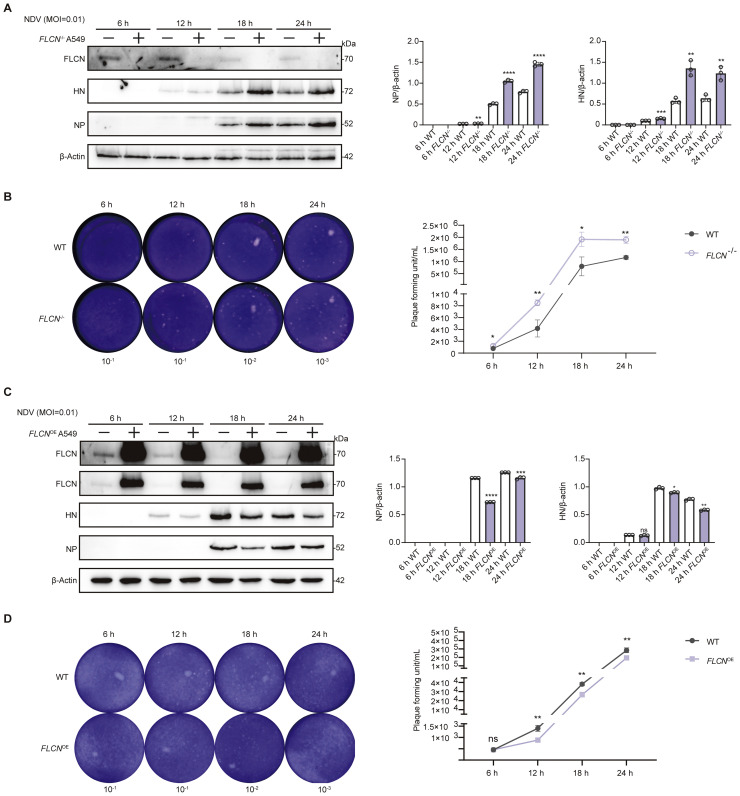
The impact of FLCN on NDV replication. A. Viral proteins NP and HN were used to indicate viral replication levels. A549 WT and *FLCN*^-/-^ cells were infected with NDV at an MOI of 0.01, and cell samples were collected every 6 hours. FLCN levels were measured to reflect the cellular FLCN content. B. The virus plaque assay was employed to determine the level of viral particles in the cell supernatant. A549 WT or *FLCN*^-/-^ cells were infected with NDV at an MOI of 0.01, and cell supernatants were collected every 6 hours. C. Viral proteins NP and HN were used to indicate viral replication levels. A549 WT or *FLCN*^OE^ cells were infected with NDV at an MOI of 0.01, and cell samples were collected every 6 hours. FLCN levels were measured to reflect the cellular FLCN content. D. The virus plaque assay was employed to determine the level of viral particles in the cell supernatant. A549 WT or *FLCN*^OE^ cells were infected with NDV at an MOI of 0.01, and cell supernatant was collected every 6 hours.

**Fig. 4:**
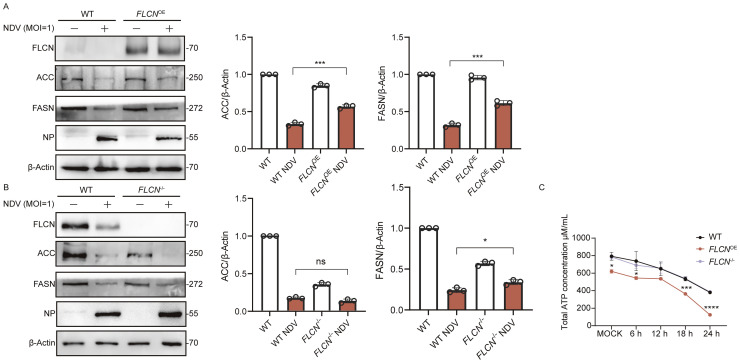
NDV infection modulates *de novo* fatty acid synthesis through FLCN and regulates energy homeostasis. A. A549 WT and *FLCN*^-/-^ cells were infected with NDV at an MOI of 1, and cell samples were collected 18 h p i. The FLCN content was utilized to identify the cell lines. B. A549 WT and *FLCN*^OE^ cells were infected with NDV at an MOI of 1, and cell samples were collected 24 h p i. The FLCN content was utilized to identify the cell lines. C. ATP levels were measured in mock-infected and NDV-infected (1 MOI) A549 WT, *FLCN*^-/-^ and *FLCN*^OE^ cells. Samples were collected 18 h p i to evaluate ATP levels.

The observation that NDV induces the degradation of FLCN, which inhibits NDV replication, suggests that NDV has developed countermeasures to overcome FLCN's antiviral effects. Notably, NDV infection suppresses the de novo fatty acid synthesis pathway while simultaneously promoting the degradation of FLCN, a positive regulator of this pathway. To further investigate whether FLCN is involved in the regulation of *de novo* fatty acid synthesis following NDV infection, we conducted additional experiments using *FLCN*^-/-^ and *FLCN*^OE^ cells. In *FLCN*^OE^ cells, NDV-induced degradation of *de novo* fatty acid synthesis-related enzymes were partially reversed (Fig. 3, Fig. 4A), whereas no such effect was observed in *FLCN*^-/-^ cells (Fig. 4B). These results reveal NDV degrades FLCN to inhibit *de novo* fatty acid synthesis. Simultaneously, we observed that the energy levels in *FLCN*^OE^ cells post-viral infection were significantly lower compared to both WT and *FLCN*^-/-^ groups. Based on these findings, we propose that NDV targets FLCN to regulate de novo fatty acid synthesis, thereby reducing unnecessary energy expenditure and facilitating viral replication.

In this study, our findings demonstrate that NDV promotes the ubiquitin-mediated degradation of FLCN to suppress de novo fatty acid synthesis. Inhibiting this biosynthetic pathway contributes to maintaining energy homeostasis in infected cells, thereby facilitating efficient viral replication.

## Discussion

Many studies have reported that different viruses employ distinct mechanisms to modulate cellular energy metabolism; for instance, dengue virus and coronavirus enhance mitochondrial β-oxidation to boost energy production (Heaton and Randall, 2010, Yousefi et al., 2022, Yue et al., 2023), while Influenza A Virus amplifies the glycolytic process to generate ATP (Ren et al., 2021, Zhang et al., 2024). These alterations often preferentially support the replication of specific viruses (Thyrsted and Holm, 2021). Increasing evidence highlights the importance of lipid metabolic remodeling during viral infection, which is often critical for efficient viral propagation (Leier et al., 2020, Farley et al., 2022). NDV has been extensively studied as an oncolytic virus (Zhong et al., 2025). Given the recognized role of fatty acids in cancer progression (Currie et al., 2013), it is crucial to investigate how NDV exploits de novo fatty acid synthesis to support viral replication in tumor cells. In this study, our transcriptomic analysis showed that NDV infection alters de novo fatty acid synthesis-related genes, reduces SREBP1c levels, and downregulates FLCN, a positive regulator of SREBP1c. FLCN degradation-induced suppression of fatty acid synthesis may promote viral replication by preserving cellular energy homeostasis.

Although NDV infection suppresses the *de novo* fatty acid synthesis pathway, this regulatory pattern is not universally observed across all viral infections. Hepatitis C virus infection frequently induces hepatic lipid accumulation, ultimately leading to the development of fatty liver disease and even cirrhosis (Ferguson et al., 2017). During HCV infection, SREBP1c is activated, subsequently upregulating the *de novo* fatty acid synthesis pathway, which plays a critical role in HCV-induced hepatic steatosis (Park et al., 2009, Meng et al., 2019). HCV relies on lipid droplets and the endoplasmic reticulum to form replication compartments (Lee et al., 2019), and the increased fatty acid synthesis-induced lipid droplet accumulation facilitates viral replication (Park et al., 2009). In addition to lipid droplet synthesis, newly synthesized fatty acids can be further utilized to produce phospholipids and other lipid species that contribute to cellular membrane formation. For instance, poliovirus requires phosphatidylcholine synthesis to establish viral replication compartments (Viktorova et al., 2018). These cases illustrate how de novo synthesized fatty acids are repurposed to support viral assembly and replication. Although fatty acids can be oxidized for energy by entering the mitochondria as acyl-CoA, using de novo fatty acid synthesis followed by β-oxidation for energy is inefficient, as synthesis consumes significant energy (acetyl-CoA, malonyl-CoA, NADPH), only to reverse the process during breakdown (Paiva et al., 2021). In contrast, direct activation of mitochondrial β-oxidation offers a more efficient pathway for energy generation, which some viruses exploit to support replication (Heaton and Randall, 2010, Yue et al., 2023). Both the inhibition of *de novo* fatty acid synthesis and the activation of fatty acid β-oxidation exert comparable contributions to maintaining cellular energy homeostasis. Our previous reports showed that NDV reprograms energy metabolism, inducing the Warburg effect and utilizing aerobic glycolysis for energy supply in tumor cells (Gong et al., 2022). Therefore, it is reasonable that NDV suppresses the *de novo* fatty acid synthesis to reduce unnecessary energy expenditure and promotes its replication. It is noteworthy that tumor cells rely on *de novo* fatty acid synthesis to support their proliferation (Koundouros and Poulogiannis, 2020), and viral suppression of this pathway highlights the potent regulatory capacity of viruses over cellular metabolism. This also partially explains the upregulation of FLCN transcription post-infection, as cells may attempt to restore fatty acid synthesis via compensatory FLCN expression. However, NDV infection counteracts this by degrading FLCN via the ubiquitination pathway, ultimately achieving suppression of fatty acid synthesis.

FLCN is a positive regulator of SREBP1c (Gosis et al., 2022); however, our transcriptomic data revealed a significant upregulation of its transcriptional levels, which contrasts with the observed downregulation of *de novo* fatty acid synthesis-related enzymes. Nevertheless, further analysis of FLCN protein levels revealed its degradation via ubiquitination following NDV infection. This pronounced discrepancy between post-infection mRNA and protein levels is likely attributable to the virus’s tight regulation of host cellular metabolism. This observation suggests that FLCN may serve as a critical target during viral replication. Consequently, we established FLCN-modified cell lines and, for the first time, validated its essential role in viral replication. It is also important to note that FLCN possesses additional functions beyond those investigated in this study, suggesting that our interpretation of its impact on viral replication may be limited in scope.

In summary, our results demonstrate that NDV infection suppresses de novo fatty acid synthesis through the ubiquitination-mediated degradation of FLCN. This transformation contributes to the maintenance of energy homeostasis in infected cells, thereby facilitating efficient viral replication. This study presents a novel mechanism by which NDV hijacks the host cell's energy metabolism system, provides new insights into the oncolytic mechanisms of NDV and offers novel perspectives for the development of tumor therapy and antiviral drugs.

## Author contributions

M.Y, conceptualization, methodology, writing—original draft, writing—review & editing; X.K, methodology, data curation; X.L, methodology, data curation; Y.S, Y.L, X.Q, L.T, and C.S, conceptualization, software, formal analysis; C.D., conceptualization, funding acquisition, validation, supervision, writing—review & editing. All authors have read and agreed to the published version of the manuscript.

## Informed consent statement

All participants provided informed consent prior to participating in the study.

## Declaration of competing interest

The authors declare no conflicts of interest.

## Appendix Supplementary materials

Image, application 1
