# Supplementary material for: Newcastle disease virus induces degradation of folliculin to regulate host cell energy metabolism and facilitate viral replication
Source: Poult Sci. 2025 May 23;104(8):105314. doi: 10.1016/j.psj.2025.105314 (PMC12164175; doi:10.1016/j.psj.2025.105314)
Supplement: Supplementary file 1 [file mmc1.zip › Supplementary figure.pptx]

## Slide 1
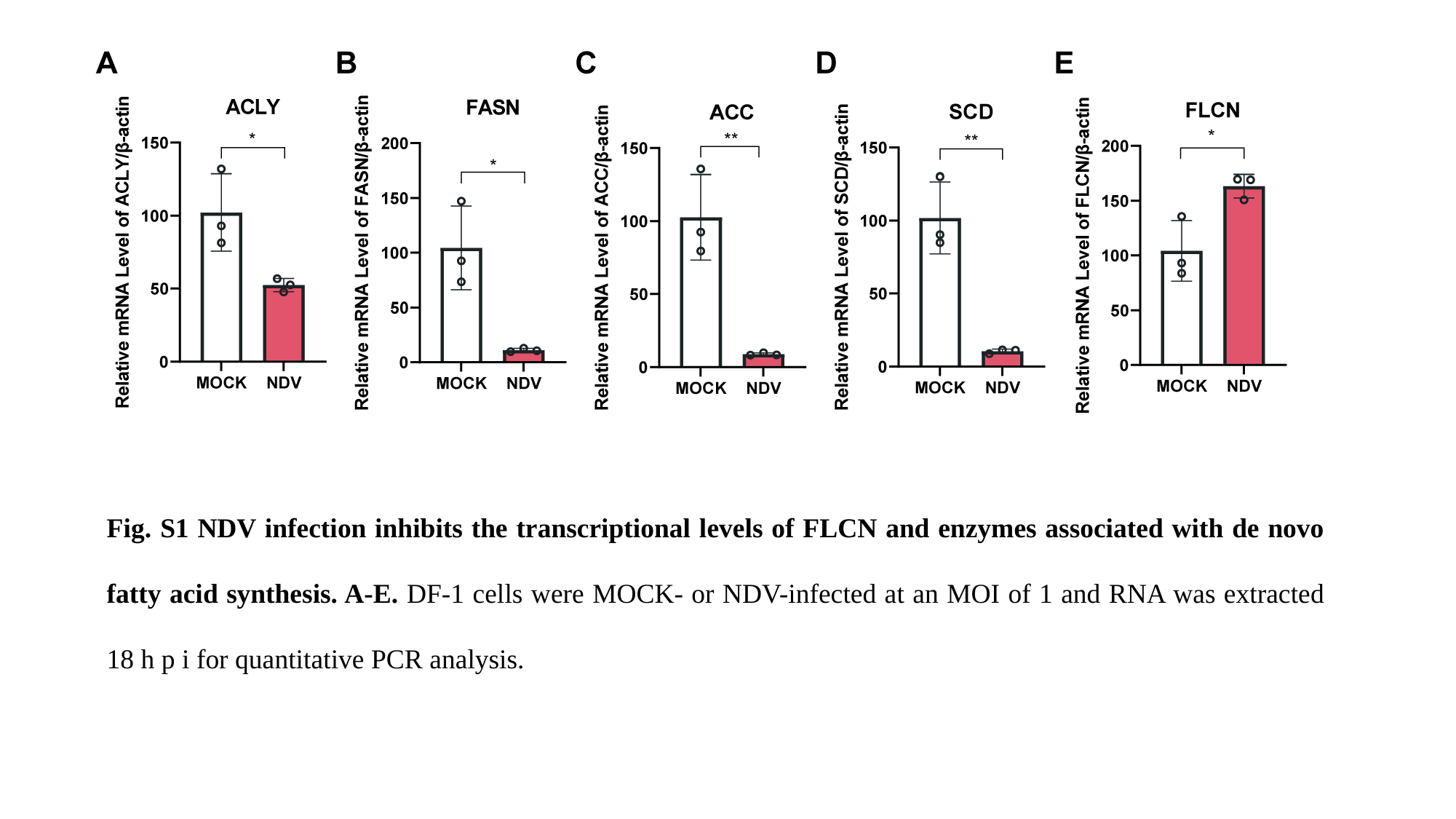

Fig. S1 NDV infection inhibits the transcriptional levels of FLCN and enzymes associated with de novo fatty acid synthesis. A-E. DF-1 cells were MOCK- or NDV-infected at an MOI of 1 and RNA was extracted 18 h p i for quantitative PCR analysis.

## Slide 2
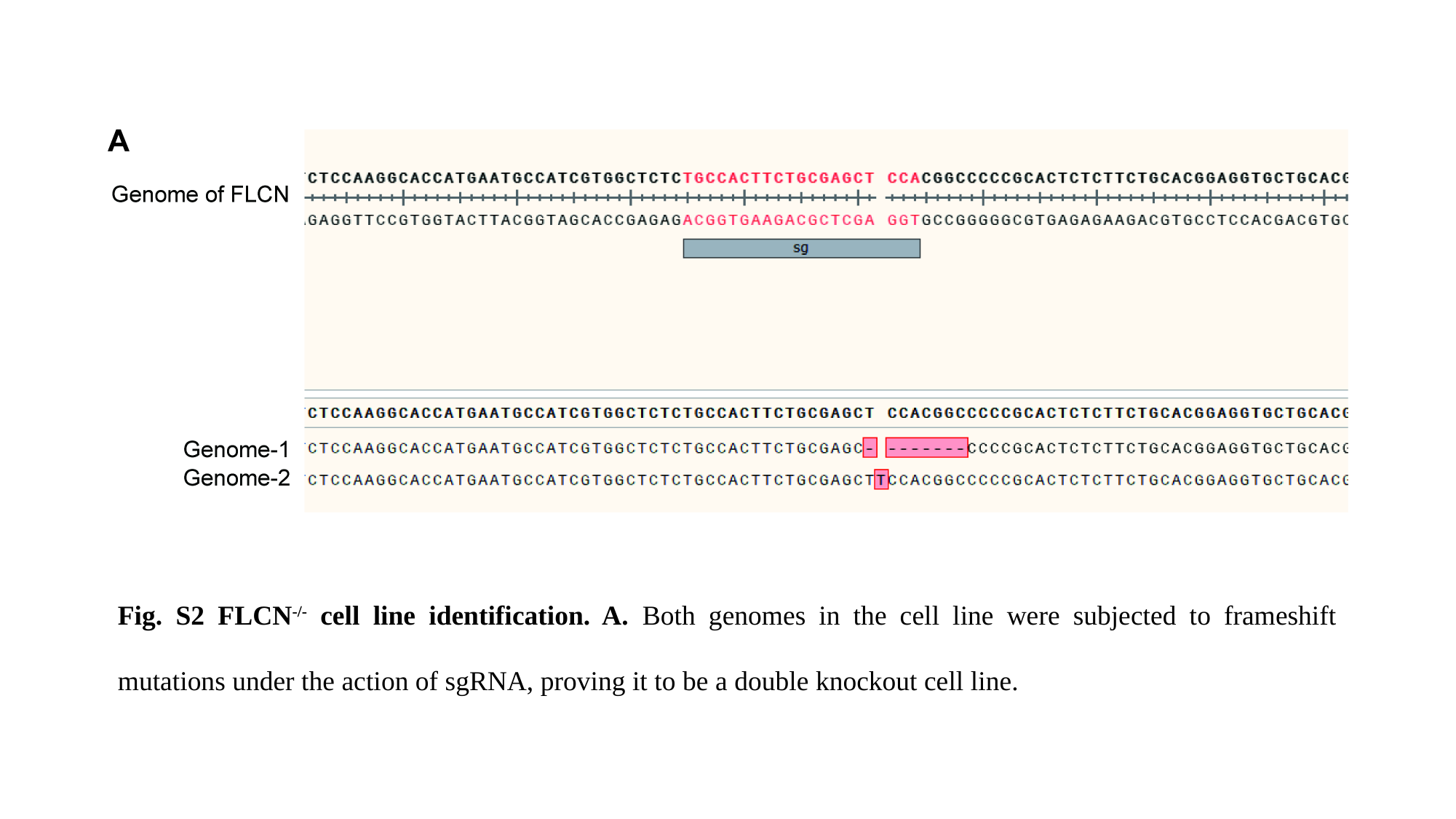

Fig. S2 FLCN-/- cell line identification. A. Both genomes in the cell line were subjected to frameshift mutations under the action of sgRNA, proving it to be a double knockout cell line.

## Slide 3
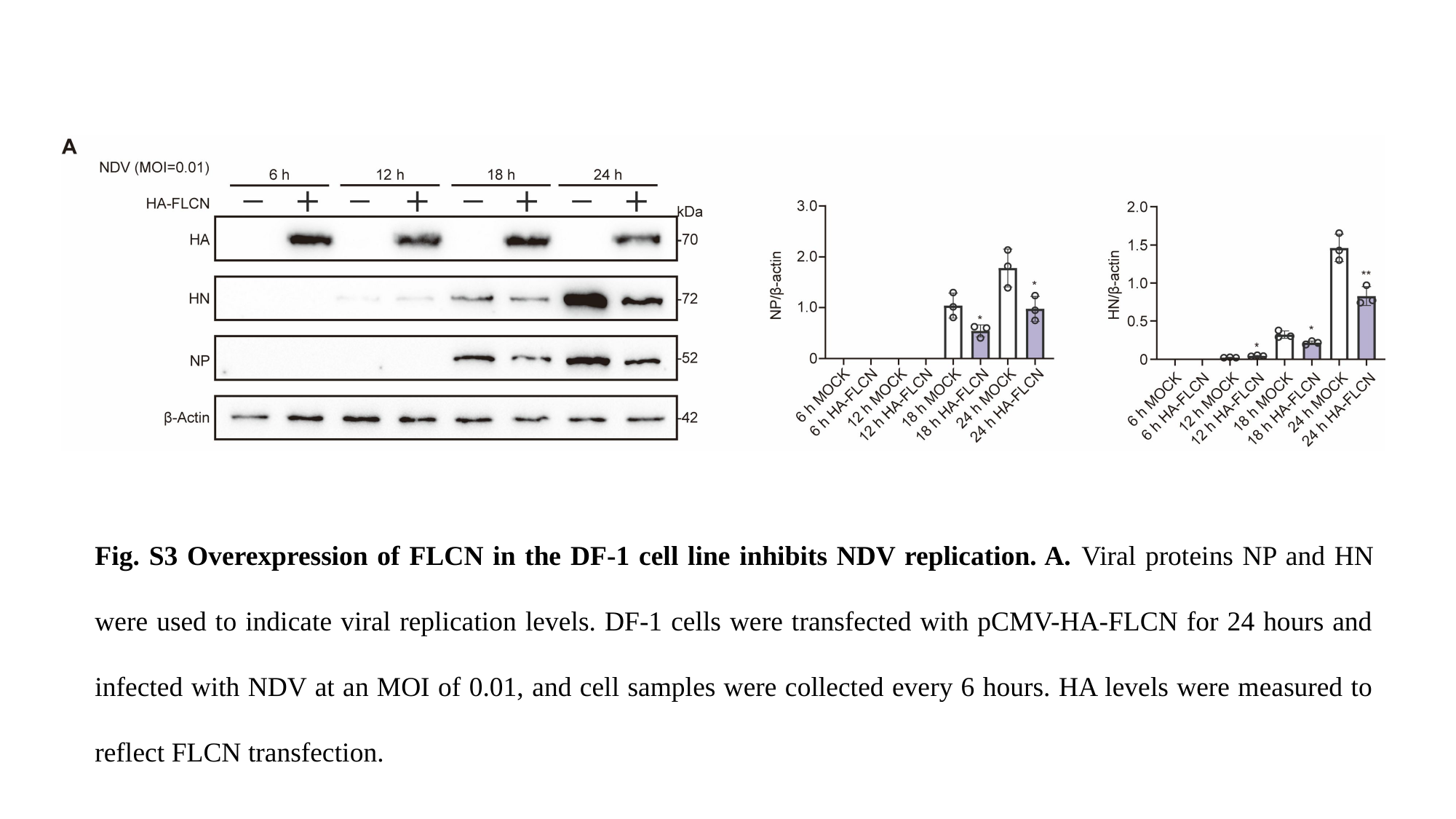

Fig. S3 Overexpression of FLCN in the DF-1 cell line inhibits NDV replication. A. Viral proteins NP and HN were used to indicate viral replication levels. DF-1 cells were transfected with pCMV-HA-FLCN for 24 hours and infected with NDV at an MOI of 0.01, and cell samples were collected every 6 hours. HA levels were measured to reflect FLCN transfection.
